# Lack of access to CDK4/6 inhibitors for premenopausal patients with metastatic breast cancer in Brazil: estimation of the number of premature deaths

**DOI:** 10.3332/ecancer.2020.1081

**Published:** 2020-07-30

**Authors:** Tomás Reinert, Rodrigo Pellegrini, Carlos Henrique Barrios

**Affiliations:** 1 Latin American Cooperative Oncology Group (LACOG), Porto Alegre, 90619-900, Brazil; 2 Oncoclinicas, Porto Alegre, 90570-020, Brazil; 3 Pontifícia Universidade Católica do Rio Grande do Sul (PUCRS) School of Medicine, Porto Alegre, 90619-900, Brazil

**Keywords:** breast cancer, drug therapy, health care disparities, drug access

## Abstract

**Purpose:**

A CDK4/6 inhibitor (CDK4/6i) combined with endocrine therapy is the standard of care for patients with hormone receptor-positive (HR+) and HER2-negative (HER2-) metastatic breast cancer (MBC). However, the incorporation of these agents into clinical practice remains challenging. This study aims to estimate the impact of the lack of access to ribociclib on mortality of premenopausal patients with MBC in Brazil.

**Methods:**

Based on published epidemiological studies and national cancer registries, we estimated the number of premenopausal patients with potential indication of ribociclib as first-line treatment for MBC. Efficacy estimates were based on results from the Monaleesa-7 trial. Our analysis is made under the unrealistic assumption that all premenopausal MBC patients would be candidates for the treatment. To estimate the number of yearly premature deaths that could be prevented, we considered the largest absolute effect on mortality when sequentially applying the observed hazard ratio.

**Results:**

We estimated an annual incidence of 4,294 premenopausal HR+, HER2- MBC patients in Brazil. Considering these patients, at 12, 24 and 60 months, the number of surviving subjects would be 3,504, 2,859 and 1,553 for endocrine therapy (ET) alone; and 3,717, 3,217 and 2,086 for ET plus ribociclib. The largest difference between both groups was observed at the end of the sixth year when the use of ribociclib would prevent 538 premature deaths (survival of 1,805 versus 1,267 patients by the 72nd month).

**Conclusion:**

We estimate that lack of access to CDK4/6i for patients with HR+, HER2-, MBC will cause the premature death of a significant number of premenopausal women with MBC. The unavailability of effective therapies has measurable consequences. Progress in this area demands a concerted effort to prevent further loss of lives.

## Introduction

Breast cancer (BC) is the most frequent malignancy in women worldwide, and it is estimated that 70% of BC deaths occur in women from low-income and middle-income countries (LMIC) [1]. Over the last few years, results of randomised clinical trials have reported significant advances in the management of different subtypes of the disease with overall survival benefits that have a definite impact in clinical practice. However, the access to these advances and the provision of high-level healthcare remains a significant challenge, particularly for LMIC. Within this scenario, it should be acknowledged that health care systems and policies are heterogeneous, and especially in LMIC, are not adequately prepared to deal with a complex problem, such as cancer [2].

Although not exclusive, one of the most important aspects of reducing cancer mortality is the availability and uptake of innovations and life-saving cancer agents [3]. The access to high-cost medications for advanced BC remains a challenging issue in several, if not most, regions of the world. Young patients with advanced BC have very particular medical, emotional and social needs. In Brazil, we have shown that young women presenting with BC have unfavourable clinicopathological features, with more aggressive tumour subtypes and advanced stage at the presentation when compared with older women [4].

In HR-positive advanced or metastatic BC (MBC), the recent introduction of CDK4/6 inhibitors (CDK4/6i) has revolutionised outcome results with significant improvements in progression-free survival (PFS) and overall survival (OS). Particularly for premenopausal patients with HR-positive MBC, endocrine therapy (ET) with the combination of ovarian function suppression, an aromatase inhibitor and the CDK4/6 inhibitor ribociclib has changed the standard of care, with the demonstration of a statistically significant and clinically meaningful survival benefit when compared to ET alone [8]. Unfortunately, access to this therapeutic strategy remains very low in our country, both in the public health system as well as in patients treated in the private setting.

It is important to recognise that this exercise is made under the unrealistic assumption that all premenopausal patients with MBC in Brazil would be candidates and have adequate criteria to be treated with the proposed drug. We acknowledge as well that is not fair to consider that all patients that start therapy in clinical practice (a completely different scenario from clinical research) will be fully compliant and derive the same or similar benefit as demonstrated in a clinical trial. Even taking these limitations into consideration with this analysis, we attempt to estimate the potential impact of the lack of access to ribociclib on the mortality of premenopausal patients with HR+ MBC in Brazil.

## Methods

To calculate the total number of young women that would be potential candidates to receive Ribociclib according to Monaleesa-7 criteria (pre- and peri-menopausal women with HR-positive, HER-2-negative MBC) we used two main sources (i) the Brazilian epidemiologic study AMAZONA-I; and (ii) the Oncology Structured Information System (OSIS) databank. The AMAZONA-I is a national epidemiologic study that followed a cohort of 634 MBC cases diagnosed in the year of 2012. It gathers data from private and public hospitals in different sites across the country [9, 10]. The OSIS database imports and merges data from Hospital Cancer Registries and from DataSUS, Agência Nacional de Saúde and Instituto Brasileiro de Geografia e Estatística (all government agencies responsible for collecting national cancer information) building a national registry of cancer. It generates not only aggregate numbers but also analyses granular data, allowing users to search for specific information such as HR and HER2 expression. This database has already been published and validated as a consistent source of cancer data in Brazil [11, 12].

In a recent publication, we addressed the prevalence and annual incidence of MBC in Brazil and estimated the proportion of HR-positive, HER2-negative cases using OSIS information [12]. To estimate the number of pre- and peri-menopausal cases, we used published data from the AMAZONA-I study. Finally, and according to the inclusion criteria of the Monaleesa-7 trial of only first-line patients, we decided to use the incidence rather than the prevalence of MBC as our population, given that we would not be able to estimate the treatments’ effect in second-line cases and beyond.

We based the OS benefits on the recently reported Monaleesa-7 results [13]. This is an international, randomised, double-blind, placebo-controlled, phase 3 trial comparing ribociclib with placebo, in addition to ET, in pre- or peri-menopausal women with HR-positive, HER2-negative advanced breast cancer. Results show a statistically significant effect (HR 0.71, 95% CI 0.54–0.95, *p* = 0.009) favouring the ribociclib cohort, with an improvement in the median OS in this population. The control group had a median OS of 40.9 months and, at a median follow-up of 34.6 months, the OS for the ribociclib group had not been reached.

We considered the results of two possible first-line treatments for these patients: (i) ET alone (control) and (ii) ribociclib plus ET. The survival distributions were measured as *S(n) -> exp(-θ.n)* for the control; and *S(n) -> exp(-r.θ.n)* for ribociclib (*r* is the HR for Ribociclib*; θ* is the hazard monthly rate and *n* is the month) [14, 15] (see Appendix for more details).

Based on the HR and the median OS of the control group, we calculated that the largest absolute effect on mortality associate with ribociclib over the control group was seen at the end of the sixth year [16]. Therefore, the absolute difference in surviving patients in the two cohorts (ribociclib minus control) at that time was considered as the total number of premature deaths to be avoided with the use of the CDK4/6 inhibitor (also see Appendix for the mathematical explanation).

We conducted a sensitivity analysis on the estimated parameters to evaluate how changes in their values modify the result. The estimation’s parameters are (i) the total candidates to receive Ribociclib (constituted by MBC incidence, the proportion of HR+HER2- and the proportion of premenopausal patients); (ii) the median overall survival of the control group and (iii) the hazard ratio for survival rates between Ribociclib and the control group.

## Results

### Estimating the population of newly diagnosed premenopausal HR-positive HER2-negative MBC in 2020 in Brazil

**Figure 1.**
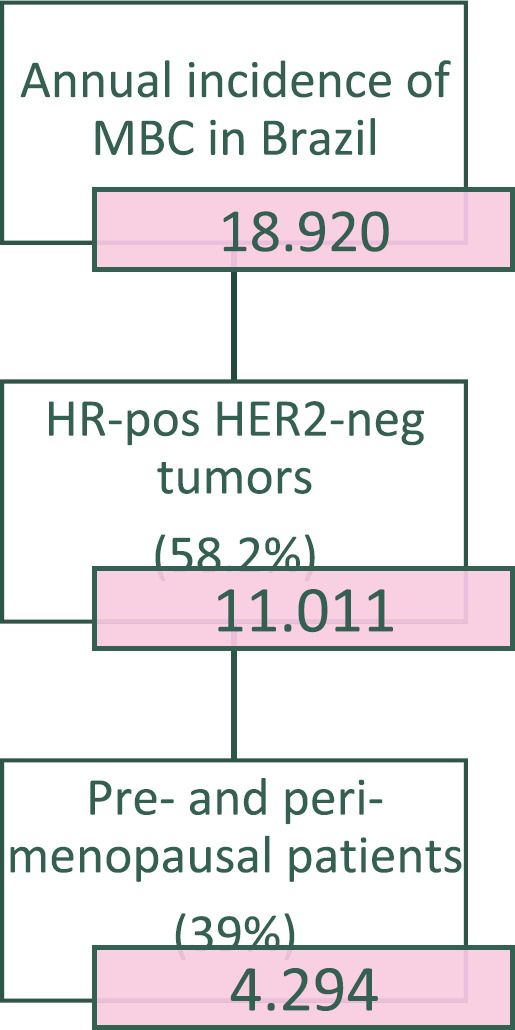
Flowchart for the estimation of the number of pre- and peri-menopausal patients from Brazil diagnosed with ER-positive HER-2-negative metastatic breast cancer in 2020.

The estimated incidence of MBC in Brazil for the year 2020 is 18,920 [12]. The molecular subtypes proportions of this total are: (i) 58.2% are HR-positive and HER2-negative (HR+ HER2-); (ii) 25.3% are HER2-positive and (iii) 16.4% are triple-negative (TN). This yields a total of 11,011 new cases of HR+ HER2- MBC for 2020. Stratifying this number by menopausal status, 39% should be considered pre- or peri-menopausal according to the AMAZONA-I trial. Therefore, as described in Figure 1, there is an estimated annual incidence of 4,294 peri- and premenopausal women with HR+, HER2- MBC that could be considered candidates for treatment with ribociclib in addition to standard ET as explored in the MONALEESA-7 study [13].

### Survival estimates

Based on the Monaleesa-7 results, we calculated a monthly mortality rate (proportion of patients that dies in each cohort) of 1.69% for the control group and 1.2% for the ribociclib group. Applying these proportions, the survival estimates in each group at 12, 24 and 60 months are 3,504 versus 3,717; 2,859 versus 3,217 and 1,553 versus 2,086, respectively. When calculating the maximum effect of ribociclib on avoiding premature deaths in this Brazilian population of pre and peri-menopausal patients with HR+, HER2- MBC, the biggest effect on survival is seen at the end of the sixth year. At this time, we estimated that 1,805 women would be alive if treated with ribociclib plus ET versus 1,267 women alive if treated with standard ET—a net increase of 538 lives (see Table 1). Considering the incidence and the benefit from both treatment options as fixed, we assume that this is the annual number of women that die prematurely due to the lack of access to ribociclib in this setting.

### Sensitivity analysis

The sensitivity analysis shows that the three parameters of the estimation have different impacts on the result (Table 2). The number of total candidates modifies the result on the same proportion of its own change. For example, a 10% decrease in the MBC incidence, in the proportion of HR+HER2- and in the proportion of premenopausal patients all have the effect of a 10% decrease in the result of premature deaths avoided. The estimated median OS for the control group has little effect on the result. The hazard ratio for survival between treatments has the highest impact on the result. Therefore, we also applied the original confidence interval of the hazard ratio from the Monaleesa-7 trial (0.54–0.95) to evaluate its impact on the result.

## Discussion

It is essential to recognise that the consequences of depriving a significant proportion of patients of effective therapy can be estimated. We demonstrate that lack of access to ribociclib will be responsible for a significant number of premature deaths of young women with MBC in Brazil.

Continuous and progressive improvement in the understanding of the complexity and heterogeneity of MBC has led to clinical trials exploring treatment strategies directed to specific subgroups of the disease. As a consequence, recently reported trials in all three major subtypes of MBC (HER2-positive, TN and HR-positive) have resulted in statistically significant and clinically meaningful benefits in OS [17–19]. Even though the disease remains mostly incurable, these advances consistently and reproducibly extend the survival of patients with metastatic disease while preserving their quality of life. Accordingly, major international guidelines have changed standard therapy recommendations for these patients [20, 21].

In patients with HR-positive HER2-negative MBC, guidelines recommended ET instead of cytotoxic chemotherapy, with the exception of cases presenting with visceral crisis or clear endocrine resistance [21]. In the last few years, a series of randomised clinical trials established the use of CDK4/6i in combination with an endocrine agent as the first-line treatment for RH+ MBC [21] Most of the trials with these agents have focused in the postmenopausal population. However, the MONALEESA-7 trial addressing specifically pre- or peri-menopausal patients clearly demonstrated a favourable outcome with the use of ribociclib with clinical and statistically significant gains in PFS and OS [8, 13].

Nonetheless, the ultimate goal of clinical research should be to improve outcomes in routine clinical practice affecting a much broader population of patients than those participating in research experiments. Recognising that controlled clinical trials necessarily require the inclusion of selected patient populations with very specific characteristics, translation of the results to the broader group of unselected patients we see in routine clinical practice remains a major objective. Arguably, in this very specific scenario, access to new drugs and technologies remains a fundamental barrier to achieve his objective.

Even though the access issue is of particular concern from a global perspective [22], it is particularly worrisome in LMIC where we encounter most of the patients with the disease and where most of the mortality is seen. A starting point, while addressing this challenge, is to assess the size of the problem.

We have estimated that Brazil has a total prevalence of 46.642 MBC women including all subtypes [12]. National income per capita is close to US$ 10,000, approximately one-sixth of the US and one-fourth of other high-income countries in Europe (Germany, France and the United Kingdom) [23]. Even though having universal coverage, the Brazilian health care system faces significant fragmentation, regional heterogeneities and resource limitations, with a very low relative expenditure on health (3.9% of GDP). Furthermore, government expenditure represents only 42% of the total health expenses. In this context, the introduction of costly new technologies represents a particular challenge. This problem can be identified in both the public and private health care scenarios [2].

The MONALEESA-7 7 trial included peri- and premenopausal patients with HR-positive, HER2-negative MBC [8]. We estimate an annual incidence of 4.295 patients with these characteristics in Brazil. The particular impact of the disease in this population has been clearly documented. Being a younger population, they have many more years of potential life lost with this incurable disease [24]. Premature deaths in this population affect women at the age of maximum productivity and income generation [25]. Furthermore, in view of their roles as mothers, wives and daughters, the direct impact of the disease and death of these young women in their family structure is colossal and cannot be easily measured [26]. Therefore, besides specific disease management needs, this population requires psychological and socio-economical support to address the full consequences of their illness.

Lack of access to innovative therapies in cancer care remains one of the most challenging issues in global oncology and has important implications for both patients and health care systems. In this analysis, we demonstrate that lack of access to ribociclib for peri- and pre-menopausal patients with HR-positive, HER2-negative MBC is associated with a significant number of premature deaths every year. That is, in the translation of trial results to clinical practice, lack of access results in patients dying sooner than they should. Furthermore, the lack of ribociclib for this population also results in a shorter PFS and consequently earlier introduction of chemotherapy with clear implications for quality of life.

Our analysis has limitations we need to address. We estimated the proportion of premenopausal patients and those presenting with *de novo* metastatic disease based on the results of the AMAZONA-I study. Moreover, other large Brazilian and international epidemiological studies and SEER data suggest reliability of our estimations [4, 9, 10, 12, 27, 28]. Furthermore, the extrapolation of clinical trial results to the general population remains a challenging exercise. Different patient characteristics as well as different management practices may impact the results. In the absence of more definitive real-world data on the use of ribociclib in premenopausal patients, we based our analysis on the efficacy results from the clinical trial. Additionally, we need to acknowledge that the access to ribociclib for premenopausal patients with advanced breast cancer is currently very low but our calculation was based on a scenario where no women with this indication in Brazil would have access for this drug. Furthermore, we use the assumption that all premenopausal patients with ER+ HER2- MBC would be good candidates for ribociclib therapy, when we know that is not always the case since a proportion of patients would be ineligible for CDK4/6 therapy due to a variety of factors, including but not only limited to impaired performance status or major organ dysfunction, whether disease-related or not. These limitations could potentially be associated with an overestimation of the number of premature deaths in our analysis. Therefore, we conducted a sensitivity analysis to evaluate how changes in factors such as number of candidates would modify the results.

Lack of access to optimal therapeutic strategies does have significant and potentially measurable consequences that can be estimated and impact the lives of patients. Here, we focus on one specific scenario and demonstrate the implications in terms of premature deaths. However, it is important to recognise that this is not only true for ribociclib in pre-menopausal HR-positive MBC patients but also applies to other therapeutic strategies and scenarios. In previous work from our group, we showed that delays in regulatory approval or access restrictions do have a significant impact on outcomes of HER2-positive MBC that can be easily estimated [27]. For patients with HER2-positive MBC, the lack of access to trastuzumab plus pertuzumab is associated with premature deaths of a significant number of these women [27].

Limited access to timely diagnosis, affordable and effective treatment and high-quality care are just some of the factors that result in observed disparities in cancer survival between countries and within countries [22]. While the complexity of the situation and the multiple factors involved are recognised, the oncology community needs actively participate in the development of feasible strategies to address possible solutions to the problem of access to innovative cancer therapies [2]. A clear and unbiased diagnosis of the situation is a good starting point. Potential strategies to address the access problem have been discussed elsewhere and include approaches to decreasing cancer medicine costs, optimising regulation and improving clinical research initiatives [2, 22, 28, 29]. Research participation, with all its caveats, is a practical solution that helps speeding drug development, fostering access and lowering the cost of drug development. The continuous support of clinical research in LMICs will certainly result in a very positive impact on cancer care [30].

## Conclusion

Lack of access to innovative cancer therapies, like CDK4/6i, is associated with a significant number of premature deaths of MBC patients. Recognising the complexity of the situation and the multiple factors that impact this scenario, the development of achievable solutions to the problem of drug access remains an unmet need. As demonstrated here, significant numbers of lives are at stake. In our view, only a combined, focused and concerted effort involving all stakeholders will be able to advance the discussion and creatively generate the urgent solutions our patients demand.

## Funding declaration

The authors declare that this publication does not have any specific financial disclosure and that there was no funding involved in this research.

## Conflicts of interest

- **Tomás Reinert:**
  - *Speaker honoraria*: Novartis, AstraZeneca, Pfizer, Libbs, Lilly, Pierre Fabre
  - *Consulting or advisory role*: AstraZeneca, Lilly, Novartis
  - *Research funding*: AstraZeneca
- **Rodrigo Pellegrino:** none
- **Carlos Henrique Barrios:**
  - *Stock and other ownership interests*: Biomarker, MedSIR, Tummi
  - *Speaker honoraria*: Novartis, Roche/Genentech, Pfizer, GlaxoSmithKline, Sanofi, Boehringer Ingelheim, Eisai
  - *Consulting or advisory role*: Boehringer Ingelheim, Roche/Genentech, Novartis, GlaxoSmithKline, Eisai, Pfizer, AstraZeneca, Libbs, MSD Oncology, United Medical
  - *Research funding*: Pfizer, Novartis, Amgen, AstraZeneca, Boehringer Ingelheim, GlaxoSmithKline, Roche/Genentech, Lilly, Sanofi, Taiho Pharmaceutical, Mylan, Merrimack, Merck, AbbVie, Astellas Pharma, Biomarin, Bristol-Myers Squibb, Daiichi Sankyo, Abraxis BioScience, AB Science, Asana Biosciences, Medivation, Exelixis, ImClone Systems, LEO Pharma, Millennium, Janssen, Atlantis Clinica, INC Research, Halozyme, Covance, Celgene, inVentiv Health
  - *Travel, accommodations, expenses*: Roche/Genentech, Novartis, Pfizer, BMS Brazil, AstraZeneca, MSD Oncology

## Figures and Tables

**Figure 2.**
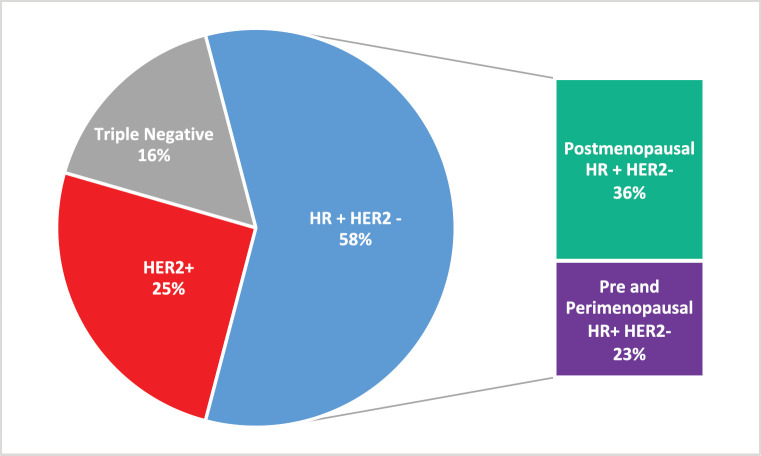
Estimated annual incidence of MBC in Brazil according to molecular subtype and menopausal status.

**Table 1.** Difference in the number of patients alive in each group as a function of the year of follow-up.

Year	Ribociclib	Control	Difference	Net effect in the last 12 months
1	3.717	3.504	213	213
2	3.217	2.859	358	145
3	2.785	2.333	452	94
4	2.410	1.904	507	55
5	2.086	1.553	533	26
6	1.805	1.267	538	5

Estimate of the survival curves for the ribociclib and the control groups. We considered a fixed HR and mortality monthly rates for both groups over time After the 72^nd^ month, the difference between groups starts to decrease. Therefore, we consider that at this point we can see the maximum effect of Ribociclib on preventing premature deaths (Column 1 represents the number of surviving patients in the ribociclib plus ET treatment arm; Column 2 represents the number of surviving patients in the ET alone arm; Column 3 is the difference of the number of surviving patients between the two groups and Column 4 represents the number of premature deaths avoided in the last 12 months).

**Table 2.** Sensitivity analysis.

Sensitivity analysis		Result if	
*Variable*	*Estimated*	*10% increase*	*10% decrease*
Candidates	18.920	592	485
Hazard ratio	0.71	390	702
mOS for control group (months)	40,9	539	538
*Variable*	*Estimated*	*Upper bound*	*Lower bound*
Hazard Ratio	0,71	81	959

Sensitivity analysis on the estimated parameters to evaluate how changes in their values modify the result. The estimation’s parameters are (i) the total candidates to receive Ribociclib (constituted by MBC incidence, the proportion of HR+HER2- and the proportion of premenopausal patients); (ii) the median overall survival of the control group and (iii) the hazard ratio for survival rates between Ribociclib and the control group.

## Appendix

Distribution

As a function of time, *x*, the event-free (survival, in this case) rates are

When, r = Hazard Ratio = Monthly hazard rate x = Time (in months)

Therefore, the benefit of the experimental treatment, as a function of time, is the difference between these rates, or .

For the estimate of the monthly hazard rate, we used the median overall survival in the control group of the Monaleesa-7 as our base (40.9 months).

Applying this result to the equation, we get that,

Maximum benefit

To determine the point of maximal separation of the survival curves(16), note that this is given by the solution to the equation of the first derivative of the Ribociclib survival minus the control group survival equals zero ->

Then,

Annual premature deaths avoided

It can be said that the maximum benefit of one treatment is equal to the annual number of premature deaths avoided. We must, however, assume that the incidence, the monthly hazard rate and the hazard ratio are constant during the period of analysis.

Here we explain:

The maximum benefit of Ribociclib, as showed above, is seen at the end of the sixth year. In this time point, 538 premature deaths are avoided in the cohort that receives Ribociclib (1.108 versus 1.267). However, we can say that this is the annual effect of Ribociclib in avoiding premature deaths if we consider that each year there are patients of different years of diagnosis (we may say different cohorts by year of diagnosis).

For example, assume that:

There are 6 cohorts of patients (2020, 2021, 2022, 2023, 2024 and 2025);

Incidence, monthly hazard rate and hazard ratio are fixed;

We are analysing data in 2026.

**Table:** Table supplementary material. Survival curves for Ribociclib and Control.

Number of patients alive				
*Month*	*Ribociclib*	*Control*	*Difference*	*Net effect in the last 12 months (Difference year(n) – Difference year(n-1))*
0 (diagnosis 2026)	4.294	4.294	0	0
12 (diagnosis 2025)	3.717	3.504	213	213
24 (diagnosis 2024)	3.217	2.859	358	145
36 (diagnosis 2023)	2.785	2.333	452	94
48 (diagnosis 2022)	2.410	1.904	507	55
60 (diagnosis 2021)	2.086	1.553	533	26
72 (diagnosis 2020)	1.805	1.267	**538**	5

In 2026, the cohort 2020 would have avoided,** during the last 12 months**, the premature death of 5 patients, since they are in the 72nd months of follow-up.

The cohort 2021, 26.

The cohort 2022, 55.

The cohort 2023, 94.

The cohort 2024, 145.

The cohort 2025, 213.

If we sum (5+26+55+94+145+213), **the result is 538.**

It is important to be noted that we used only 6 years because, after the 72^nd^ month of follow-up, the difference in survival in the cohort of Ribociclib minus the control starts to decrease. It means that there cannot be seen anymore the effect of premature deaths avoided.

Example for the year of 2027:

**Table:**

Number of Patients Alive				
*Month*	*Ribociclib*	*Control*	*Difference*	*Net effect in the last 12 months (Difference year(n) – Difference year(n-1))*
0 (diagnosis 2027)	4.294	4.294	0	0
12 (diagnosis 2026)	3.717	3.504	213	213
24 (diagnosis 2025)	3.217	2.859	358	145
36 (diagnosis 2024)	2.785	2.333	452	94
48 (diagnosis 2023)	2.410	1.904	507	55
60 (diagnosis 2022)	2.086	1.553	533	26
72 (diagnosis 2021)	1.805	1.267	**538**	5
84 (diagnosis 2020)	1.562	1.034	**528**	-5

With this example of 2027, we explain that the effect of avoiding premature deaths cannot be seen anymore, since there is a negative net effect of cohort 2020. This is the importance of considering the premature deaths, since MBC is not a curable disease.
